# 

**Published:** 1878-08

**Authors:** 


### CONTENTS:

ARTICLE I.—Offerings. By J. F. P. Hodson, D. D. S. .......... 145
ARTICLE II.—Strengthening Rubber Dentures and Repairing Broken Plates. By F. E. Howard, D. D. S. .......... 157
ARTICLE III.—Diagnostic Value of the Soft Palate, as Compared with the Tongue, in certain Pathological Conditions. By Wm. Abram Love, M. D. .......... 161
ARTICLE IV.—Mechanical Dentistry. By C. S. Butler, D. D. S. .......... 168
ARTICLE V.—Substance of Lectures on Gold (Continued.) By Prof. Hodgkin .......... 173
ARTICLE VI.—Thymol .......... 177
ARTICLE VII.—Remarks on Dr. Reynolds' Case of Restoration after Necrosis, and Removal of Inferior Maxilla. By J B. Hodgkin, D. D. S. .......... 179
American Academy of Dental Science—Change of Time of Meeting. 182

### EDITORIAL, ETC.

English Dentistry and the Chemists .......... 182

A New Blow-Pipe .......... 184

Conventions, Associations, Recreation .......... 185

### MONTHLY SUMMARY.

Rules for Chloroform Administration .......... 185
Antiseptics have no Action on the Blood .......... 186
Dangers of Horseback Exercise .......... 186
Opium vs. Coffee .......... 187
Absorption of Tincture of Iodine by the Skin .......... 187
Thymol as an Antiseptic .......... 188
Iodoform as a Local Application .......... 188
New Method of Plugging .......... 189
To Utilize Gutta-Percha Scraps .......... 189
The Physiological Action of Aconite .......... 189
Action of Chloroform on the Bladder .......... 189
Extirpation of Superior Maxilla .......... 190
Malformation of the Skull in Epileptics .......... 190
Hydrophobia Cured by Oxygen .......... 190
Vegetable Parasites .......... 191
Lead-Poisoning by Flour .......... 191
Antidote to Carbolic Acid .......... 191
Hypodermic Injections of Chloroform .......... 192
Chloroform Narcosis Cured by Nitrite of Amyl .......... 192
Disadvantage of Salicylic Acid as a Dentifrice .......... 192

